# Data on thrombotic ischemic lesions in the presence or absence of amyloid ß-protein precursor or its homolog amyloid precursor-like protein-2 in mice

**DOI:** 10.1016/j.dib.2015.11.050

**Published:** 2015-12-03

**Authors:** Feng Xu, William E. Van Nostrand

**Affiliations:** Departments of Neurosurgery and Medicine, Stony Brook University, Stony Brook, New York, USA

## Abstract

Amyloid ß-protein precursor (AßPP) and amyloid precursor-like protein-2 (APLP2) are potent inhibitors of thrombosis, see related article “The influence of the amyloid ß-protein and its precursor in modulating cerebral thrombosis” (Van Nostrand, 2016) [1]. Datapresented are images of photo-induced thrombotic ischemic stroke in wild-type mice, AßPP^−^^/^^−^ mice and APLP2^−^^/^^−^ mice, and the calculated infarct volume show approximately 40% and 33%, respectively, larger cerebral infarcts compared to wild-type mice.

**Specifications Table**

**Table:** 

Subject area	*Biology*
More specific subject area	*Thrombosis and ischemic stroke*
Type of data	*Figure, histological image, histogram*
How data was acquired	*Photo-induced thrombosis, histological staining, microscopic and stereological analysis*
Data format	*Analyzed and raw data*
Experimental factors	*AßPP*^−^^/^^−^*mice, APLP2*^−^^/^^−^*mice and wild-type mice*
Experimental features	*Photo-induced thrombotic ischemic stroke was performed in mice, brains were assessed for stroke volume by histological staining and quantitative stereological analysis*
Data source location	*Stony Brook, New York, USA*
Data accessibility	*Data are within this article*

Value of the data

• The data contains information on biological functions of AßPP and APLP2.
• The data show that the absence of AßPP or APLP2 significantly increases the severity of thrombotic ischemic stroke.
• The data indicate that both AßPP and APLP2 participate in regulating thrombotic ischemic stroke.

## 1 Data, experimental design, materials and methods

### 1.1 Photo-induced cerebral ischemic stroke in mice

We performed experimental ischemic stroke in wild-type mice and mice deficient for the AßPP gene (AßPP^−^^/^^−^) or the APLP2 gene (APLP2^−^^/^^−^) that were obtained from Jackson Laboratories (Bar Harbor, ME) to determine the influence of the absence of these proteins on thrombotic infarct volumes. All work with animals followed National Institutes of Health guidelines and was approved by the Stony Brook University Institutional Animal Care and Use Committee. Here 3 months old mice (*n*=10 animals per genotype) were subjected to photo-induced cerebral ischemic stroke to introduce a permanent focal lesion in the cortex of one brain hemisphere as described [2], [3]. Briefly, the anesthetized mouse was placed in a stereotaxic apparatus and an aseptic surgical area was washed and draped. The scalp was shaved and an incision area was prepared with alcohol and iodine (Betadine). The mouse internal temperature was monitored throughout the surgery using a rectal thermistor (Barnant Company, Barrington, IL) and was maintained at 37±0.5 °C with the use of a heating pad. A sagittal incision was made caudal to rostral allowing the scalp to be retracted and held in place with micro-clips to expose the skull surface. A helium neon laser beam (Melles Griot, Carlsbad, CA), was focused on the skull 1.5 mm posterior and 2 mm paramedian from the bregma. Then 0.1 cc of the photoactivated dye Rose Bengal (50 mg/kg in 0.9% saline) was injected through the tail vein and the skull was simultaneously exposed to the neon laser beam. The beam intensity was fixed at 1.5 mW, 543.5 nm, for a duration of 15 min. Following laser exposure, the scalp was closed under sterile conditions using 4-0 nylon suture. The animal was placed in a cage warmed with a heating pad and observed until it is alert and mobile. All animals that have undergone surgery were given a dose of Buprenophine (0.05–0.01 mg/kg) s.q. post-operatively with additional doses given as needed.

### 1.2 Histological analysis and infarct volume measurement

**Fig. 1:**
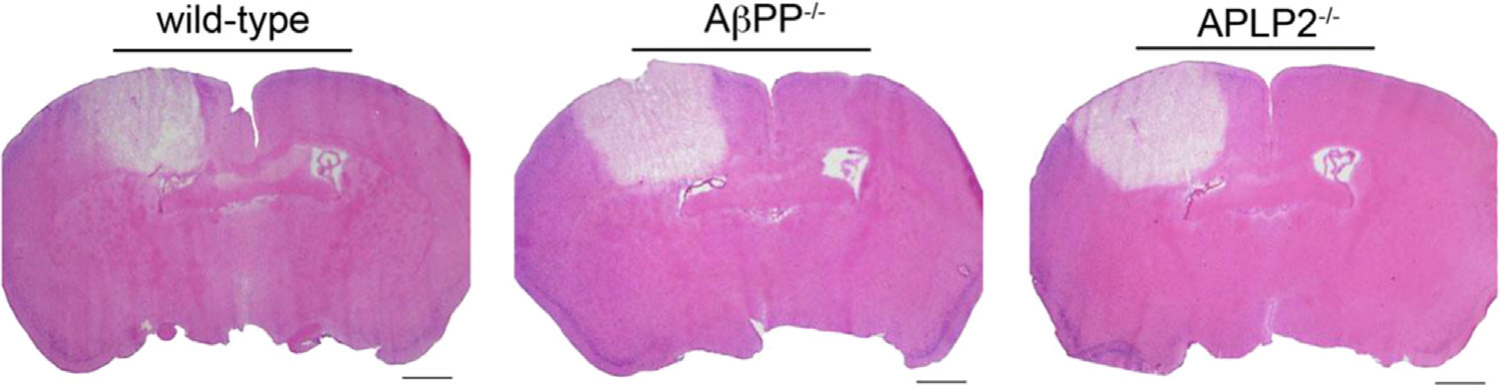
Representative images of photo-induced thrombotic ischemic lesions in wild-type, AβPP^−/−^ and APLP2^−/−^ mice.

**Fig. 2:**
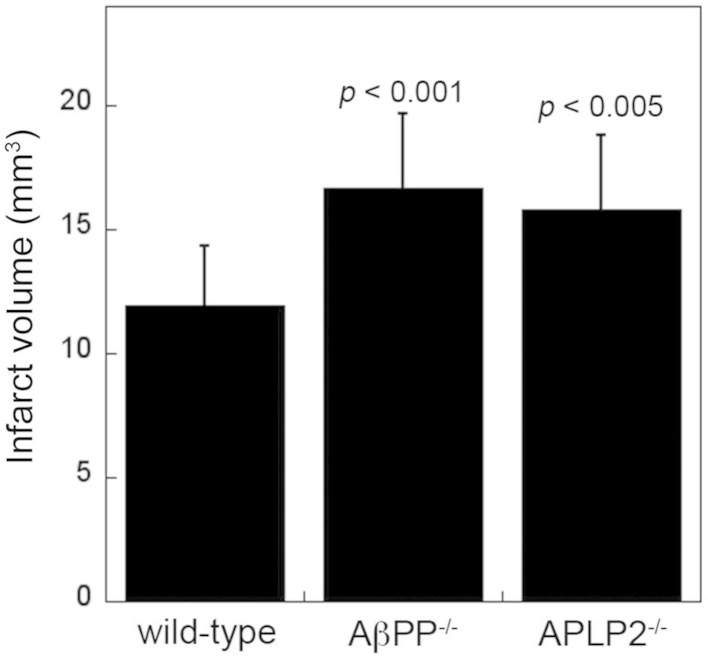
Quantitation of infarct volumes in wild-type, AβPP^−/−^ and APLP2^−/−^ mice.

Following the cerebral ischemic stroke (24 h) the mice were sacrificed and perfused with phosphate-buffered saline. The brains were harvested, embedded in OCT compound (Sakura Finetek Inc., Torrance, CA) and then frozen at −80 °C. The frozen brain tissue was cut coronally in 20 µm sections, every 10th section was collected (approximately 8–10 sections per brain spanning the infarct) and mounted on glass slides. Sections were stained with Hematoxylin and Eosin Y (Fig. 1). An Olympus BX60 microscope with a digital camera was used to capture images. The infarct volume was measured using the Stereologer software system (Systems Planning and Analysis, Inc. Alexandria, VA). Compared with wild-type mice the infarct volumes in AβPP^−/−^ mice were ≈40 larger (*p*<0.001) and ≈33% larger in APLP2^−/−^ mice (*p*<0.005) (Fig. 2).

### 1.3 Statistics

Data were analyzed by student׳s *t* test at the 0.05 significance level.
