# Supplementary material for: Data on thrombotic ischemic lesions in the presence or absence of amyloid ß-protein precursor or its homolog amyloid precursor-like protein-2 in mice
Source: Data Brief. 2015 Dec 3;6:149–52. doi: 10.1016/j.dib.2015.11.050 (PMC4706567; doi:10.1016/j.dib.2015.11.050)
Supplement: Supplementary file 1 — Supplementary material [file mmc1.docx]

Conflict of Interests

The authors have no conflicts of interest to declare.
